# Dual-functionalized architecture enables stable and tumor cell-specific SiO_2_NPs in complex biological fluids

**DOI:** 10.3762/bjnano.15.100

**Published:** 2024-10-07

**Authors:** Iris Renata Sousa Ribeiro, Raquel Frenedoso da Silva, Romênia Ramos Domingues, Adriana Franco Paes Leme, Mateus Borba Cardoso

**Affiliations:** 1 Institute of Chemistry (IQ), University of Campinas (UNICAMP), Postal Code 13083- 970, Post Office Box 6154, Campinas, SP, Brazil; 2 Brazilian Synchrotron Light Laboratory (LNLS), Brazilian Center for Research in Energy and Materials (CNPEM), Postal Code 13083-970, Campinas, Brazil; 3 Brazilian Biosciences National Laboratory (LNBio), Brazilian Center for Research in Energy and Materials (CNPEM), Postal Code 13083-970, Campinas, Brazil

**Keywords:** colloidal stability, complex media, functionalized nanoparticles, hemolysis, targeting tumor

## Abstract

Most commercial anticancer nanomedicines are administered intravenously. This route is fast and precise as the drug enters directly into the systemic circulation, without undergoing absorption processes. When nanoparticles come into direct contact with the blood, however, they interact with physiological components that can induce colloidal destabilization and/or changes in their original biochemical identity, compromising their ability to selectively accumulate at target sites. In this way, these systems usually lack active targeting, offering limited therapeutic effectiveness. In the literature, there is a paucity of in-depth studies in complex environments to evaluate nanoparticle stability, protein corona formation, hemolytic activity, and targeting capabilities. To address this issue, fluorescent silica nanoparticles (SiO_2_NPs) are here functionalized with zwitterionic (kinetic stabilizer) and folate groups (targeting agent) to provide selective interaction with tumor cell lines in biological media. The stability of these dually functionalized SiO_2_NPs is preserved in unprocessed human plasma while yielding a decrease in the number of adsorbed proteins. Experiments in murine blood further proved that these nanoparticles are not hemolytic. Remarkably, the functionalized SiO_2_NPs are more internalized by tumor cells than their healthy counterparts. Investigations of this nature play a crucial role in garnering results with greater reliability, allowing the development of nanoparticle-based pharmaceutical drugs that exhibit heightened efficacy and reduced toxicity for medical purposes.

## Introduction

In recent years, there has been a growing search for developing high-efficiency nanomedicines for cancer treatment [1–4]. Consequently, the scientific community has focused on improving the targeting of nanoparticles (NPs) to tumor cells through surface functionalization with active groups (e.g., folate, monoclonal antibodies) [5–7]. However, according to the literature, only 0.7% of the injected dose of NPs accumulates in tumors and <0.0014% are internalized by the cells [8–11]. Once in contact with blood, NPs interact with a series of physiological components (e.g., amino acids, salts, and proteins), which can induce poor colloidal stability or changes in the original chemical and biological identity of these particles, impairing their therapeutic efficiency [12–15]. Proteins and other biomolecules can be adsorbed on the surface of NPs (protein corona formation), masking their original functionality and hiding their target ability [16–18]. Protein corona can further lead to the formation of aggregates of NPs and activation of unplanned biological pathways (e.g., the complement system) [16–18]. The factors mentioned above hamper the biodistribution of nanomedicines and their targeting efficiency, therefore causing adverse effects.

Multiple functionalization strategies to produce colloidally stable NP dispersions with retained targeting capacity have been widely investigated [17–21]. While a panel of molecules (e.g., polyethylene glycol, zwitterionics, glycans, aptamers) can improve the stability of NPs in biological media by preventing the adsorption of proteins [22–23], developing methods that can meet the trade-off between targeting and colloidal stability of NPs in such media has proven to be a major challenge [8,10–11]. Our group produced SiO_2_NPs functionalized with a steric stabilizer (zwitterionic sulfobetaine silane) and biologically active groups (amino, mercapto, or carboxylic functionalities) to interact with cells, viruses, and bacteria [17–18,20]. Pan et al. [24] developed mesoporous silica nanoparticles (SiO_2_NPs) covalently attached to (i) poly(oligo(ethylene glycol) monomethyl ether methacrylate) for improved stabilization and (ii) targeting peptide for targeted delivery aimed at increasing efficiency against cancer. Although valuable, these systems have not been able to provide reduced protein corona formation and targeting ability, or they have not been scrutinized in complex biological environments. In fact, there is a lack of more in-depth studies in complex media to assess the stability of NPs, along with the possible protein corona formation, eventual hemolytic activity, and targeting ability. Parameters such as the length of stabilizing and directing groups and their absolute concentrations and proportions play a key role in obtaining NPs with the desired stability and functionality in bodily fluids [12].

Here, we proposed developing SiO_2_NPs colloidally stable in biological media and targetable to tumor cells through selective binding with folate receptors, highly expressed in tumor cell lines. Sulfobetaine zwitterionic (ZW) and folate groups were chosen as kinetic stabilizers and targeting agents, respectively. It is worth highlighting that sulfobetaines were selected specifically for their enhanced hydration properties, effectively preventing protein adsorption on various NPs and contributing to improved colloidal stability [25–26]. Remarkably, functionalized NPs were stable in a complex medium (cell culture medium and human plasma) and showed greater potential for recognition by tumor cells.

## Material and Methods

### Materials

Tetraethyl orthosilicate (TEOS, 98%), (3-aminopropyl)triethoxysilane (APTES, 99%), ammonium hydroxide (NH_4_OH, 28%), rhodamine B isothiocyanate, 1-ethyl-3-carbodiimide hydrochloride (EDC), *N*-hydroxysuccinimide (NHS), and folic acid were purchased from Sigma-Aldrich. (*N*,*N*-Dimethylaminopropyl)trimethoxysilane (DMAPTMS), 1,3 propanesultone (99%), ethyl acetate (HPLC), (3-aminopropyl)triethoxysilane (99%) were purchased from Fisher Scientific. Ethanol (absolute) was purchased from Merck. All reagents and chemicals were used as received without further purification. Water used in the described procedures was obtained from a water purification system (Purelab from ELGA, resistivity of 18.2 MΩ·cm^–1^).

### Synthesis of rhodamine-labeled SiO_2_NPs (SiO_2_NPs)

The synthesis of rhodamine-labeled SiO_2_NPs was similar to the protocol proposed by the group [27]. The dye precursor was synthesized by conjugation of the isothiocyanate group of rhodamine B isothiocyanate to APTES at a molar ratio of 50:1 (dye: APTES) in absolute ethanol under nitrogen. The resulting solution was continuously stirred for 24 h. The conjugate was then hydrolyzed in a basic ethanolic solution with TEOS and catalyzed by ammonia. In summary, the dye precursor (0.1 mL) was added to ethanol (120.0 mL), TEOS (2.0 mL), and ammonia solution (6.1 mL), and the resulting mixture was stirred for 3 h. Afterward, the conjugate and TEOS were added at the same concentrations as described above, and the reaction was stirred overnight. The NPs were centrifuged (10000 rpm, 15 min), followed by a single wash with ethanol and four washes with deionized water. Finally, they were re-dispersed in 120.0 mL of water. After, a silica shell was synthesized around the preformed fluorescent core by the addition of ethanol (60.0 mL), ammonia solution (1.4 mL), and TEOS (1.7 mL). The addition of TEOS was done at a rate of 1.0 mL per minute, using a syringe pump (New Era Pump Systems, NE8000, Farmingdale, NY). Posteriorly, the solution remained under stirring for 24 h. The resulting suspension was purified and re-dispersed in water for storage and subsequent functionalization.

### Functionalization of SiO_2_NPs

Before the functionalization of SiO_2_NPs, the synthesis of the ZW compound was performed. This synthesis was adapted from the protocol proposed by Litt et al. [28] Briefly, 5.0 g of DMAPTMS were added in a two-neck round bottom flask containing 10.0 mL of dry ethyl acetate. The temperature was stabilized at 45 °C and the flask was placed under stirring. Subsequently, 3.0 g of 1,3-propane-sulfone diluted in 20.0 mL of dry ethyl acetate was added. The solution was brought to a temperature of 50 °C, remaining under stirring for 3 h. Then, the ZW compound was purified by centrifugation and resuspension process four times in acetone. The compound was dried using a vacuum pump and stored in a desiccator. The product was analyzed by proton nuclear magnetic resonance (^1^H NMR) in deuterated water (D_2_O, 600 μL) with the standard trimethylsilyl propionic acid (TMSP). The signal referring to the solvent used, D_2_O, was omitted from the spectra as shown in Supporting Information File 1, Figure S1.

The SiO_2_NPs were functionalized following the proportion of 95% ZW:5% folate. These percentages were defined based on previous studies carried out by the group [18,20,29]. For functionalization, 30.0 mL of the freshly prepared NP solution (without purification) was transferred to a flask kept in a bath at 80 °C. Then, 26.0 mg of ZW, previously solubilized in 2.0 mL of water, was added. The solution was stirred and heated for 4 h. Subsequently, the heating was removed and 0.98 µL of APTES was added. The reaction was stirred for 24 h. The purification process was carried out by successive washings by centrifugation, using ethanol and water. The addition of the tumor driver to the NPs was performed by activating the carboxyl groups of folic acid with the use of EDC, in the presence of NHS, and subsequent reaction with amine groups present in the NPs. The amount of folic acid used was the same as the number of moles of APTES.

The chemical structures of the main compounds used for this process are represented in Supporting Information File 1, Figure S2. The NPs were named as: SiO_2_NPs (non-functionalized), SiO_2_NPs-ZW (with ZW), SiO_2_NPs-ZW-NH_2_ (with ZW + APTES), and SiO_2_NPs-ZW-FO (with ZW + APTES + folate).

### Characterization of SiO_2_NPs

Scanning electron microscopy (SEM) micrographs were obtained in a high-resolution FEI Inspect F50 microscope. A NP suspension (7 μL) was deposited directly onto a copper substrate, dried, and sputter-coated with Au using a Bal-Tec SCD050 Sputter Coater. Secondary electrons were collected after backscattering of the Au-coated samples attained by electron beams with a 5 kV acceleration voltage.

The particle hydrodynamic diameter and zeta potential were evaluated on a Malvern Zetasizer ZS equipment (Malvern Instruments Ltd., UK – detection angle of 173° and laser wavelength of 633 nm). For DLS measurements, NPs were dispersed in MilliQ water (1.0 mg·mL^–1^). To determine the zeta potential, the NPs were diluted in 10 Mm of phosphate buffer at a concentration of 1.0 mg·mL^–1^. All measurements were made in triplicates at room temperature.

The ^1^H NMR spectra were obtained in Bruker spectrometers operating at frequencies of 500 MHz. The NP dispersions (25 mg) were centrifuged and concentrated to 600 μL of D_2_O with the standard TMSP. The samples were sonicated before measurements. Chemical shifts (δ) were related in part per million (ppm) to the standard TMSP. The signal referring to the solvent used, deuterated water, was omitted from the spectra.

The elemental analysis measurements were performed on a CHN 2400 Elementary Analyzer (Perkin-Elmer). The NP dispersions were dried in an oven at 100 °C and the percentages of carbon, nitrogen, and hydrogen were obtained. It is noteworthy that only the nitrogen percentages were considered for the calculations.

The elemental composition of the NP surface was obtained using a K-Alpha XPS (Thermo Fisher Scientific) which operates with Al Kα X-rays with charge compensation. Spectra were recorded in three distinct areas per sample with 400 μm spatial resolution, using 200 eV pass energy. High-resolution spectra for C 1s, N 1s, Si 2p, and S 2p were recorded with a resolution of 0.1 eV, using a pass energy of 40 eV. All spectra were analyzed using CasaXPS^®^ software.

Immunoprecipitation was used to characterize the functionalization of NPs. A schematic of the magnetic beads preparation can be seen in Supporting Information File 1, Figure S3. Magnetic beads (50 μL) at a concentration of 30 mg·mL^–1^ (Dynabeads MyOne Carboxylic Acid, Thermo Fisher Scientific) were modified by adding 20 mg of folate receptor alpha protein (FRα, pro-2162 Recombinant Human Folate Receptor 1, Synapse Biotechnology). Incubation was carried out at room temperature for 40 min, under manual agitation. Subsequently, a magnet was used to capture the beads with the adsorbed protein, and the supernatant was discarded. The bead+protein set was then incubated with 50 mM (hydroxymethyl)aminomethane buffer (Tris, pH 7.4) + 0.03% bovine serum albumin (BSA) + 0.1% Triton X for 60 min at room temperature and under manual agitation. Then, the supernatant was removed, and the SiO_2_NPs and SiO_2_NPs-ZW-FO (8.9 mg·mL^–1^) were added such that the amount of folate receptor was twice that of SiO_2_NPs-ZW-FO. The incubation was carried out for 3 h at room temperature, so the supernatant was collected and analyzed. The samples were measured using the fluorescence technique (Tecan Infinite Microplate Reader equipment). The excitation wavelength was set at 524 nm and the emission spectra were generated in the spectral window from 560 to 700 nm with a constant gain value of 120. The analytical curve was obtained from the determination of several dilutions of non-functionalized SiO_2_NPs (0.1 to 1.0 mg·mL^–1^) and later used for the quantification of captured SiO_2_NPs-ZW-FO. The calculations were performed using the value obtained at the maximum of the emission band.

### Stability of SiO_2_NPs in cell culture medium and human plasma

Dynamic light scattering (DLS) measurements were performed to evaluate the colloidal stability of NPs in complex media. The media used for these measurements were i) Dulbecco’s modified eagle medium (DMEM, Sigma-Aldrich), supplemented with 10.0% fetal bovine serum (FBS, Sigma-Aldrich), with 1.0% of non-essential amino acids (Sigma-Aldrich), and with 1.0% of penicillin and streptomycin (Sigma-Aldrich) and ii) human plasma. The human plasma was in compliance with ethics in research committee (CEP) of UNICAMP/Brazil. Particularly, the plasma was diluted (final percentage of 5.5%) in phosphate-buffered saline (PBS, pH 7.4, 10 mM) for incubation with particles. The plasma concentration was determined based on in vitro experiments found in the literature using animal blood [30–31]. In such experiments, mouse blood is diluted in PBS to obtain a concentration of 10 mg·mL^–1^ of hemoglobin. Generally, to achieve this concentration the blood must be diluted to a concentration of 10%. In this case, as the concentration of plasma in the blood is 55% [32–33], the plasma must be diluted to 5.5% to carry out the experiments. Analyses were performed in triplicate at 37 °C, with incubation times of 0, 1, 2, 3, and 24 h. The NP concentration used was 0.5 mg·mL^–1^.

### Protein corona formation in cell culture medium and human plasma

To verify the adsorption of proteins on the functionalized SiO_2_NPs, sodium dodecyl sulfate polyacrylamide gel electrophoresis (SDS-PAGE) experiment was performed. For this, the nanomaterials (in concentrations of 2 to 5 mg·mL^–1^) were incubated in DMEM culture medium (10% FBS) at 37 °C for 10 min, using the thermoblock Thermomixer C (Eppendorf). Posteriorly, the precipitate was washed with PBS (pH 7.4, 10 mM) to remove excess proteins weakly adsorbed on SiO_2_NPs. The washing procedure was performed by centrifugation (14000 rpm for 10 min at 4 °C) and was repeated four times. Then, Laemmli buffer (1610737, Bio-Rad) containing 50 mM of dithiothreitol (DTT, 1610611, Bio-Rad) was added to the final precipitate, followed by heating at 95 °C for 5 min, and 10 μL of this suspension was applied in a 12% SDS-PAGE gel. The gel was run at a voltage of 200 V for 1 h and then the gel was stained with silver nitrate to visualize the bands. PageRuler™ Prestained Protein Ladder (Thermo Scientific) was used as a standard. The DMEM culture medium used for SiO_2_NP incubation was also added to the gel for comparison purposes.

To determine the total protein concentration present in the protein corona of each NP, bicinchoninic acid (BCA) assay was used, following the recommendations of the manufacturer (23225, Thermo Scientific). First, the functionalized and non-functionalized SiO_2_NPs (concentration of 3 mg·mL^–1^) were incubated in i) DMEM culture medium (10% FBS) and ii) human plasma (5.5% in PBS) at 37 °C for 10 min, using the thermoblock Thermomixer C (Eppendorf). After this period, the precipitate was washed with PBS (pH 7.4, 10 mM) to remove excess proteins weakly adsorbed on the surface of SiO_2_NPs. The washing procedure was performed by centrifugation (14000 rpm for 10 min at 4 °C) and repeated four times. The SiO_2_NPs were resuspended in 25 μL of water and transferred to a 96-well plate. Then, 200 µL of working reagent (50:1, reagent A/B) was added. Finally, the plate was incubated at 37 °C for 30 min and quantification was performed using the absorbance value at 562 nm, using the Thermo microplate reader (Multiskan GO model). For quantification, it was necessary to prepare an analytical curve with diluted BSA standards already included in the kit provided by the manufacturer. Analyses were performed in quintuplicate.

To identify the main proteins adsorbed on the SiO_2_NPs, liquid chromatography coupled with mass spectrometry (LC-MS/MS) was used. The proteins were separated in SDS-PAGE as described limiting the protein run to only enter in the beginning of the resolving gel. Then, the gel was stained with Coomassie brilliant blue, and the unique bands from each sample were cut out. The preparation of samples for analysis by LC-MS/MS was performed according to previously described protocols [34–35]. Briefly, the protein bands were bleached with a destaining solution and dehydrated in acetonitrile. Reduction and alkylation of disulfide bridges were performed with dithiothreitol and iodoacetamide, respectively, followed by in-gel trypsin digestion.

The peptides were desalted on a C18 Stage Tips column and an aliquot containing 2 µg of each sample was analyzed using an LTQ Orbitrap Velos (Thermo Fisher Scientific) coupled to a nanoflow EASY-nLC (Proxeon Biosystems) liquid chromatography system through a Proxeon nanoelectrospray ion source. Peptides were separated at a flow rate of 300 nL/min in a 2–90% acetonitrile gradient in 0.1% formic acid for a total gradient time of 65 min (35% acetonitrile at 33 min) using a PicoFrit analytical column (20 cm × ID75, 5 μM particle size, New Objective). The source temperature was set to 275 °C and the nanoelectrospray voltage to 2.2 kV. The mass spectrometer operated in data-dependent acquisition mode, where full scan MS1 spectra (*m*/*z* 300–1,600) were acquired at resolution *r* = 60,000 after accumulation of 1 × 10^6^ ions. The 20 most intense peptide ions with charge state ≥2 were sequentially isolated to a target value of 5000 and fragmented by collision-induced dissociation in the linear ion trap using a normalized collision energy of 35%. Dynamic exclusion was enabled with an exclusion size list of 500 peptides, duration of 60 s, and repetition count of 1.

**Proteome Data Analysis:** Raw data was processed using MaxQuant v1.5.8 22 and protein identification was performed against the UniProt Human Protein Database (97,511 protein sequences, released in 2021) and the Bos taurus Database (6,404 protein sequences, released in 2022) using the Andromeda search engine. Carbamidomethylation of cysteines (+57.02 Da) was set as fixed modifications, and protein N-terminal acetylation (+42.01 Da) and methionine oxidation (+15.99 Da) were set as variable modifications. Trypsin/P was set as the proteolytic enzyme, with a maximum of two missed cleavages allowed. A tolerance of 10 ppm for precursor ions and 1 Da for fragment ions was defined. A maximum of 1% FDR calculated using reverse sequences was set for both protein and peptide identification.

### Cell viability assays

HaCat and KB cells were cultured in a DMEM culture medium and kept in an atmosphere of 5% CO_2_, and 95% air at 37 °C in a humidified incubator. Subsequently, cells were seeded onto 96 well microplates at a density of 1 × 10^4^ cells/well at a final volume of 200 μL and incubated for 24 h to obtain a subconfluent monolayer. Cells were exposed to SiO_2_NPs at concentrations of 0.05, 0.10, 0.50, and 1.00 mg·mL^–1^. After 24 h of incubation, cells were washed three times with PBS 1×, and Alamar Blue (Invitrogen, 10% in DMEM) was added to each well. The samples were incubated for 3 h at 37 °C. The supernatant was then collected from the wells, put in a fresh 96-well plate, and analyzed with a spectrophotometer (EnSpire 2300, Perkin Elmer) setting the excitation wavelength at 560 nm and the emission wavelength at 590 nm. Analyses were performed in triplicate.

### Hemolysis assay in human concentrated red blood cell plastic bag and murine blood

Transfusion service human concentrated red blood cell (RBC) (type B+) plastic bags, collected according to their consent form for blood donation, were provided by the Hemocenter from the Faculty of Medicine at the University of Campinas, São Paulo, Brazil. First, the RBCs were washed three times with PBS (pH 7.4, 10 mM) followed by centrifugation at 5000 rpm for 10 min at 4 °C (centrifuge Eppendorf, 5810R model) and supernatant removal. All hemolytic assays were prepared using a stock suspension of 10% RBCs (10 mL, in PBS). Then, a PBS solution was used to dilute functionalized and non-functionalized SiO_2_NPs at concentrations ranging from 0.5 to 1.0 mg·mL^–1^. Later, 100 μL of the RBC stock suspension was added to distinct tubes and incubated in the static method for 1 h at room temperature after gentle homogenization. The final volume of the hemolytic assay in all experiments was 1 mL. Then, all tubes were centrifuged (10 min) and 200 μL of supernatant was removed from each tube and transferred to a 96-well plate. The supernatant hemoglobin quantification was done by measuring the absorbance of the solution (540 nm) with a microplate reader (Thermo, Multiskan GO model). Deionized water (900 μL) and RBC stock suspension (100 μL) were used as a positive control, whereas PBS solution (900 μL) and RBC stock suspension (100 μL) were used as a negative control. All hemolytic tests were performed in triplicate. A similar assay was performed but the NPs were incubated in DMEM (10.0% FBS) and not in PBS.

The ability of the particles to cause hemolysis was also evaluated using mouse blood. For these experiments, ten Swiss mice were used, obtained from the Multidisciplinary Center for Biological Investigation of the State University of Campinas (CEMIB/ UNICAMP). All animals obtained from CEMIB received water and food ad libitum (Nuvilab) and were kept in the vivarium of the Department of Cellular and Structural Biology (Area of Anatomy) of the Institute of Biology, under the responsibility of Dr. Valéria Helena Alves Cagnon Quitete, until they reached the experimental age for euthanasia. At 2 months of age, the animals were weighed on a Denver P-214 analytical balance (Denver Instrument Company), anesthetized with 2% xylazine hydrochloride (5 mg·kg^–1^, intramuscular; König) and 10% ketamine hydrochloride (60 mg·kg^–1^, intramuscular; Fort Dodge) and had their blood collected by cardiac puncture, followed by euthanasia of the animal. The procedures were approved by the Ethics Committee on Animal Use (protocol 5725-1/2021) and conducted in accordance with the Ethical Principles for Research with Animals, established by the Brazilian College of Animal Experimentation (COBEA). The detailed experimental procedure is described in ASTM standard E2524-08 (Standard test method for analyzing the hemolytic properties of nanoparticles) [30,36]. Briefly, the total hemoglobin concentration of heparinized blood was measured using the cyanomethemoglobin method (EIAHGBC, Thermo Scientific), based on an analytical curve of hemoglobin concentration at an absorbance wavelength of 540 nm. The blood was then diluted to a hemoglobin concentration of 10 mg·mL^–1^ with Ca^2+^/Mg^2+^-free PBS. Functionalized and non-functionalized SiO_2_NPs were evaluated at concentrations of 0.5, 1.0, 3.0, and 5.0 mg·mL^–1^. Aliquots were prepared for a final volume of 1 mL, considering 80 µL of blood and the remainder NP + PBS. The tubes were incubated in an oven at 37 °C for 180 min, with gentle inversion of the sample tubes every 30 min. After incubation, the tubes were centrifuged at 10000 rpm for 10 min (4 °C), and aliquots of the supernatant (200 μL) were carefully removed from the tubes and transferred to a 96-well plate. The quantification of hemoglobin in the supernatant and the preparation of positive and negative controls were performed as described above.

### Folate receptor expression and internalization of SiO_2_NPs in healthy and tumor cells

To verify the folate receptor expression, the Western blot technique was used, which consists of transferring proteins from a polyacrylamide gel to an adsorbent membrane [37]. The identification of the target protein is performed through the specific recognition of a specific secondary antibody.

To identify the folate receptor in KB (high receptor expression) and HaCat (low receptor expression) cells, they were initially washed with ice-cold PBS (pH 7.4, 10 mM), followed by the addition of approximately 1 mL of cell lysis buffer (RIPA with protease inhibitor (50×)). Afterward, they were incubated on ice for 30 min and homogenized at predetermined times. Next, the samples were sonicated for 10 s and subsequently centrifuged (12000 rpm, 20 min, 4 °C). The supernatant was recovered, kept on ice, and quantified using the BCA assay. All measurements were performed simultaneously and in triplicate. After quantification of total proteins in the cell lysate, 20 μg of total protein per sample was mixed with Laemmli buffer with DTT (50 mM) and separated by an SDS-PAGE assay. Proteins were transferred to a nitrocellulose membrane (Bio-Rad transfer system) using transfer buffer (2.5 mM TrisHCl, 20 mM glycine, 0.01% SDS and 20% methanol). Transfers were performed for 1 h 30 min (100 V). Next, the membranes were incubated with blocking buffer (Tris saline solution with Tween 20 (TBS-T) + 3% BSA) for 1 h (room temperature). Subsequently, they were washed with TBS-T buffer and incubated with primary antibody anti-folate alpha receptor at a dilution rate of 1:500 for 12 h at 4 °C. The membranes were rewashed with TBS-T buffer and incubated with a secondary antibody (Goat anti-Mouse IgG (H + L) HRP, ThermoFisher) at a ratio of 1:20000 for 2 h (room temperature). After a new series of washes with TBS-T, the peroxidase activity was revealed with a chemiluminescent solution (Clarity™ Western ECL Substrate – 1705060, Bio-Rad) for 5 min. Antibody to β-actin (AC-15, Novus) was used as an endogenous control for all samples.

To assess the internalization of NPs, HaCat and KB cells were cultured in a 96-well plate (1 × 10^4^ cells/well) for 24 h. Subsequently, the SiO_2_NPs (functionalized and non-functionalized) were dispersed in DMEM (10% FBS) and added to the cells (0.5 mg·mL^–1^). The incubation was carried out for periods of 3, 6, 12, and 24 h. Afterward, the medium with SiO_2_NPs was discarded and the cells were washed 3 times with PBS. Finally, the cells were fixed with 4% paraformaldehyde for 20 min and permeabilized with 0.1% Triton X-100 and 2% bovine serum albumin for 40 min. F-actin was stained for 30 min with 10 µg/mL phalloidin-FITC (fluorescein isothiocyanate), and the nuclei were counterstained with 2 µg/mL of 4’,6-diamino-2-phenylindole (DAPI). After that, samples were analyzed on the Operetta High Content Screening System microscope (PerkinElmer Life Sciences).

To obtain quantitative information on the internalization of functionalized and non-functionalized SiO_2_NPs, the flow cytometry technique was used. HaCat and KB cells were cultured in a 6-well plate (200,000 cells per well) for 24 h. Subsequently, the SiO_2_NPs (functionalized and non-functionalized) were dispersed in DMEM (10% FBS) and added to the cells (0.5 mg·mL^–1^). Incubation was carried out for periods of 3 and 24 h. Thereafter, the medium with SiO_2_NPs was discarded, and the cells were washed 3 times with PBS and removed from culture plates by trypsinization. Next, the cells were centrifuged to remove trypsin and subsequently washed with PBS. Following that, 4% paraformaldehyde was added for 20 min. Finally, the cells were centrifuged again, washed with PBS, and resuspended in PBS.

### Statistical analysis

The Shapiro–Wilk test evaluated the normality of data. The Student *t*-test was used to make comparisons between two sets of data with a normal distribution (parametric data), while the Mann–Whitney test was used to make comparisons between two non-parametric data sets. One-way and two-way analysis of variance (ANOVA) and Tukey’s test were used to compare three or more parametric data. Differences were considered significant when *p* < 0.05. All statistical calculations were carried out using the R program version 3.6.2 (R Development Core Team, 2019).

## Results and Discussion

### Nanoparticle characterization

**Figure 1:**
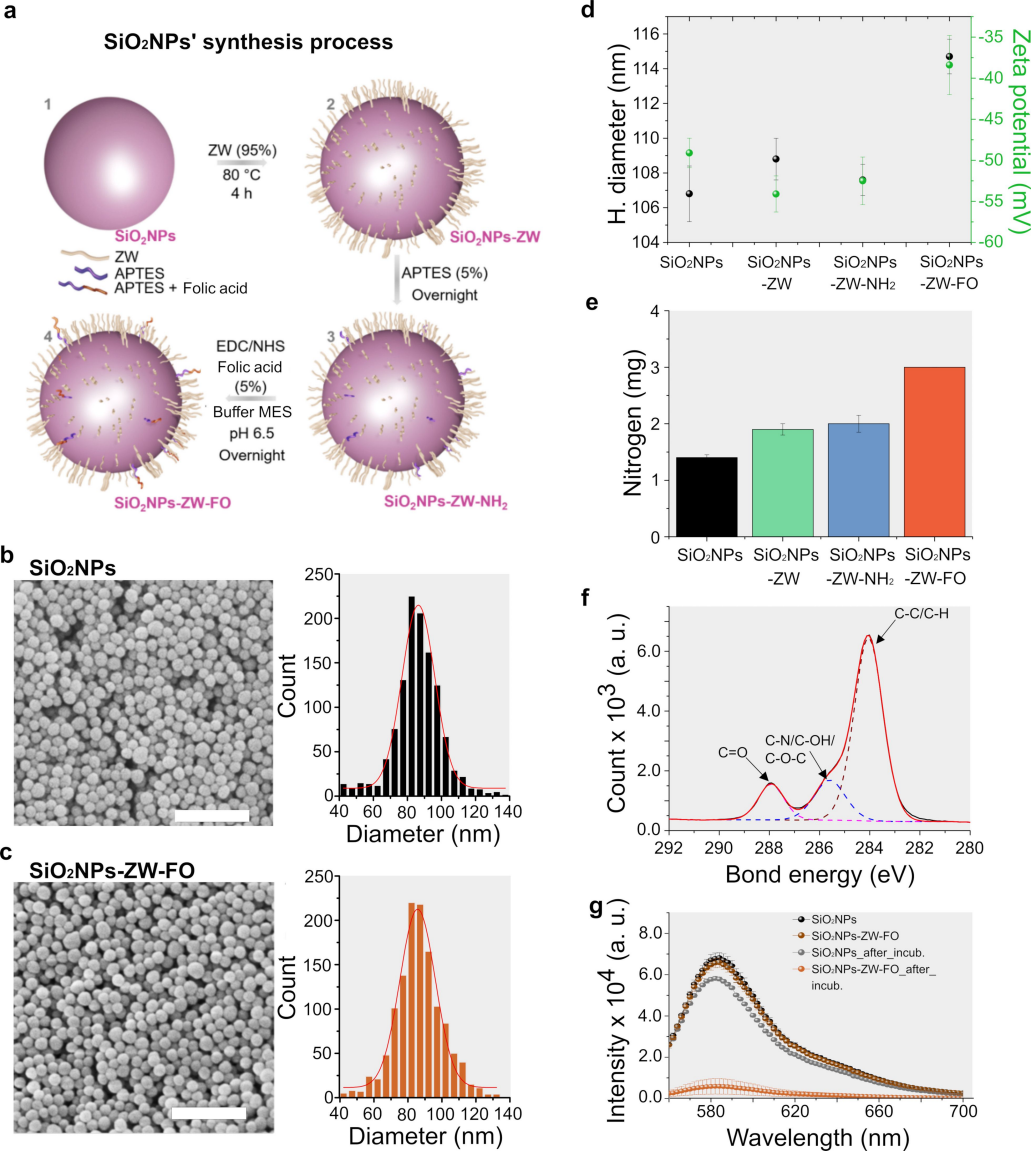
a) Representation of the synthesis steps of NPs with kinetic stabilizer and tumor driver. SiO_2_NPs: NPs without functionalization; SiO_2_NPs-ZW: NPs with zwitterionic; SiO_2_NPs-ZW-NH_2_: NPs with zwitterionic + APTES and SiO_2_NPs-ZW-FO: NPs with zwitterionic + APTES + folate. b,c) SEM image and size distribution for SiO_2_NPs and SiO_2_NPs-ZW-FO (*n* ≈ 1000), respectively. Scale bar: 500 nm. d) DLS and zeta potential results for SiO_2_NPs, SiO_2_NPs-ZW, SiO_2_NPs-ZW-NH_2_, and SiO_2_NPs-ZW-FO samples. e) Results obtained by the elemental analysis technique. Values in mg of nitrogen present in 1 g of sample for each step of the synthesis. f) High-resolution XPS spectrum (C 1s) for SiO_2_NPs-ZW-FO. The main peaks for each sample are marked on the spectrum itself. g) Fluorescence intensity for non-functionalized SiO_2_NPs and ZW-FO-SiO_2_NPs after incubation with magnetic beads coated with the folate receptor.

The functionalized NP synthesis was performed using zwitterionic as a kinetic stabilizer and folate as a tumor driver (Figure 1a). They presented a quasi-spherical shape (Figure 1b,c) and an average diameter of 86.4 ± 0.4 nm for non-functionalized SiO_2_NPs and 86.1 ± 0.5 nm for the SiO_2_NPs-ZW-FO. The DLS measurements provided the hydrodynamic diameter of SiO_2_NPs and SiO_2_NPs-ZW-FO, indicating values of 106.8 ± 1.6 nm and 114.7 ± 1.0 nm (Figure 1d), respectively. In both cases, the polydispersity index (PDI) was less than 0.08, suggesting a homogeneous size. The increase in the hydrodynamic diameter for the SiO_2_NPs-ZW-FO may be associated with their subtle aggregation after the addition of folate or simply by the change in the dynamic structure of the particle since folate is hydrophobic [35] and can alter the arrangement of water molecules around the NP.

Zeta potential measurements indicated negative charges for the SiO_2_NPs. This charge is justified by the silanol groups on the surface that are deprotonated at pH values above 4 [13,38–40]. After the addition of ZW (SiO_2_NPs-ZW), theoretically, the zeta potential value should approach zero since silanol groups would not be available to contribute to the surface charge and the ZW structure has a net neutral charge, which does not interfere with the NP charge. The maintenance of the negative charge can be explained by the existence of remaining silanol groups on the surface of SiO_2_NPs, resulting from a heterogeneous coating of the surface, and by the premature hydrolysis of the methoxyl groups of the ZW [39]. This hydrolysis can lead to the formation of silanol groups that contribute to the NP final charge [39]. Furthermore, according to the zeta potential results, no difference between SiO_2_NPs-ZW and SiO_2_NPs-ZW-NH_2_ samples was visualized. The low concentration of the functional groups was probably not significant enough for detection. The SiO_2_NPs-ZW-FO showed a less negative zeta potential value than the previous functionalization steps, which is opposite to what was expected after adding folate [38,41]. In a neutral medium, the exposed group of the folate molecule can be found in its acidic (absence of charges) and basic forms (negative charge), which should favor the increase of negative charge of the system (Figure 1d) [38,41]. However, the decrease in value can suggest a subtle increase in the degree of SiO_2_NPs-ZW-FO aggregation or even the possibility of folate hiding some negative charges on the NP surface. In short, it was impossible to confirm the steps of the SiO_2_NP functionalization process through zeta potential measurements.

Elemental analysis and XPS techniques were further used to address these limitations. The elemental analysis technique showed that the nitrogen concentration increases in the following sequence SiO_2_NP<SiO_2_NP-ZW<SiO_2_NP-ZW-NH_2_<SiO_2_NP-ZW-FO (Figure 1e). Such increase follows the synthesis proposal, since with the addition of ZW, one nitrogen is added to the SiO_2_NPs; with the addition of APTES, there is an increase of one more nitrogen, and after the insertion of folate, six more nitrogen atoms are added to the structure of SiO_2_NPs. According to this technique, the overall amount of nitrogen for ZW, APTES, and folate on the surface of SiO_2_NPs were 32 mmol·g^–1^, 11 mmol·g^–1^, and 12 mmol·g^–1^, respectively. In percentage (in moles), these values correspond to 73% ZW and 17% APTES and folate. Such results were satisfactory since they showed that the composition was similar to the stoichiometric proportion used during the synthesis, 95% ZW and 5% APTES and folate.

According to the XPS measurements, no peaks were identified in the non-functionalized SiO_2_NPs (Supporting Information File 1, Figure S4a). On the other hand, two peaks were observed for SiO_2_NPs-ZW (Supporting Information File 1, Figure S4b), which correspond to the N^+^(CH_3_)_3_ (402 eV) and N–C (400 eV) bonds, coming from the ZW group and from remnants of ZW precursors, respectively. For SiO_2_NP-ZW-NH_2_ and SiO_2_NP-ZW-FO (Supporting Information File 1, Figure S4c, S4d), it was verified a peak referring to the N–H bond (399 eV) from both APTES and folate groups. The C 1s spectra for SiO_2_NP-ZW-NH_2_ and SiO_2_NP-ZW-FO showed the presence of peaks referring to the C-SO_3_^−^ (286 eV) and C–N^+^ (288 eV) bonds, both coming from the ZW molecule (Supporting Information File 1, Figure S4d). Furthermore, with the addition of folate, a peak referring to the C=O binding (288 eV) was verified (Figure 1f).

Finally, the immunoprecipitation test was performed to confirm the functionalization of SiO_2_NPs-ZW-FO and evaluate their recognition by the folate receptor by monitoring the fluorescence of the supernatant. Once the NPs are recognized by the receptor, the fluorescence signal of the supernatant is decreased, which was verified in greater intensity for the SiO_2_NPs-ZW-FO (Figure 1g). This reduction indicates that the functionalization process was successful and that folate remained active and could bind to the receptor. Based on the analytical curve (*R*^2^ = 0.9997 and sensitivity = 73800.9, arbitrary fluorescence units per mg·mL^–1^, Supporting Information File 1, Figure S5), only 1.1 mg·mL^–1^ of non-functionalized SiO_2_NPs were removed from the dispersion while 8.0 mg·mL^–1^ of SiO_2_NPs-ZW-FO were captured by the beads with this receptor.

### Nanoparticle colloidal stability and protein corona formation

**Figure 2:**
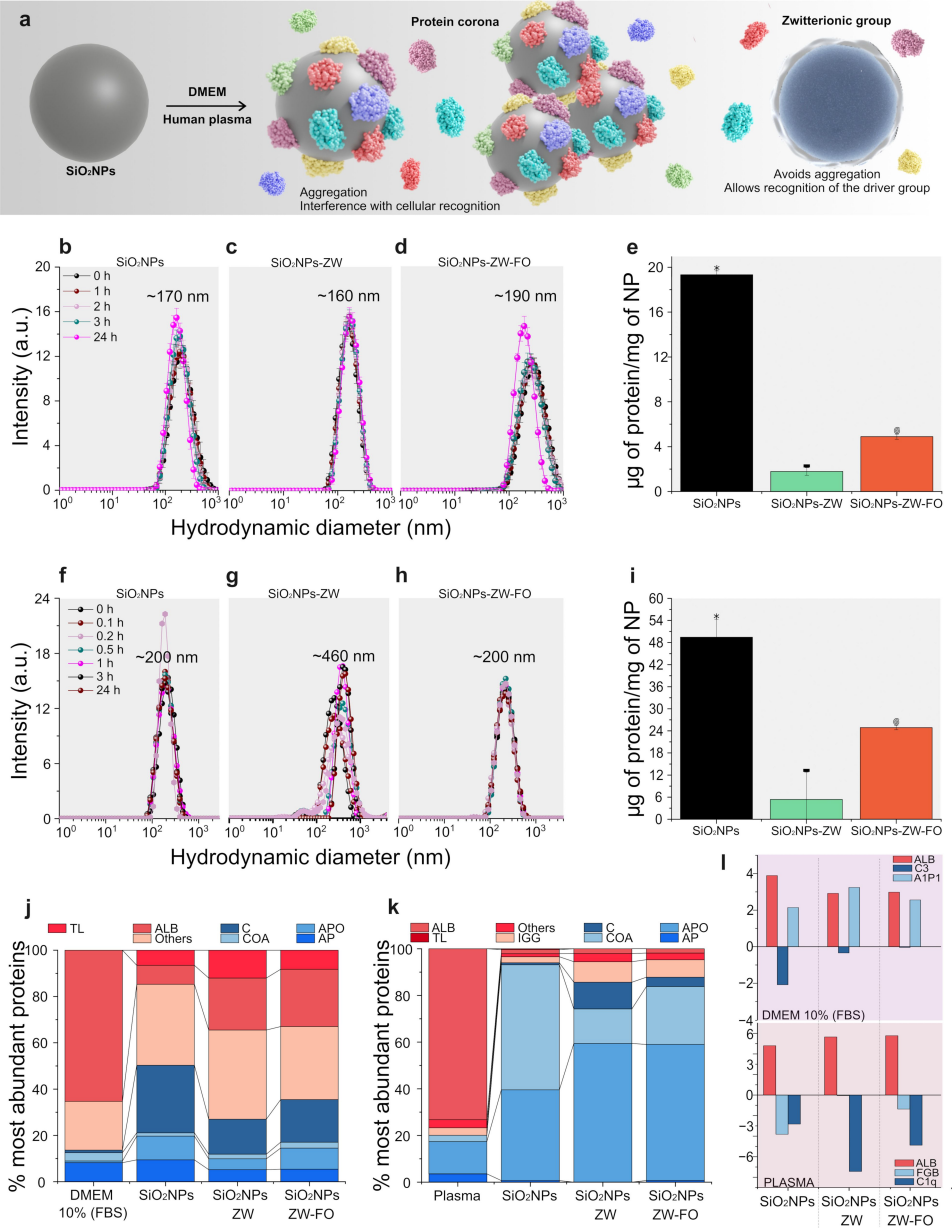
(a) Representation of the protein adsorption on the surface of NPs, which can lead to particle aggregation as well as impair their targeting efficiency. Size distribution curves for (b) SiO_2_NPs, (c) SiO_2_NPs-ZW, and (d) SiO_2_NPs-ZW-FO in DMEM (10% FBS). For this analysis, a particle concentration of 0.5 mg·mL^–1^ was used, and different incubation times were evaluated. The approximate PDI for the NPs incubated in DMEM was 0.15. (e) Quantification of proteins adsorbed on SiO_2_NPs, SiO_2_NPs-ZW, and SiO_2_NPs-ZW-FO per mg of NP after incubation in DMEM medium (10% FBS). Results are displayed as mean ± standard deviation (*n* = 5). Size distribution curves for (f) SiO_2_NPs, (g) SiO_2_NPs-ZW, and (h) SiO_2_NPs-ZW-FO in human plasma. For this analysis, a particle concentration of 0.5 mg·mL^–1^ was used, and different incubation times were evaluated. The approximate PDI for the NPs incubated in plasma was 0.18 for SiO_2_NPs and SiO_2_NPs-ZW-FO, and 0.38 for SiO_2_NPs-ZW. (i) Quantification of proteins adsorbed on SiO_2_NPs, SiO_2_NPs-ZW, and SiO_2_NPs-ZW-FO per mg of NP after incubation in DMEM medium (10% FBS). Results are displayed as mean ± standard deviation (*n* = 5). The distinct symbols at the top of the bars indicate that there is a statistical difference between them. The most abundant proteins identified by LC-MS/MS on the hard corona of non-functionalized and functionalized SiO_2_NPs in (j) DMEM (10% FBS) and (k) human plasma. (l) Statistical analysis of protein enrichment (negative numbers) and depletion (positive numbers) on SiO_2_NPs, SiO_2_NPs-ZW, and SiO_2_NPs-ZW-FO in relation to DMEM and human plasma. ALB: serum albumin, TL: tissue leakage, C: complement, COA: coagulation, APO: apolipoprotein, AP: acute phase, IGG: immunoglobulin, A1P1: alpha-1-antiproteinase, FGB: fibrinogen beta chain, C1q: complement component 1q.

One of the main challenges in nanomedicine is maintaining the NP physicochemical integrity when exposed to physiological environments, preventing protein corona formation (Figure 2a) [15–18]. It is expected that the ZW on the NP surface minimizes the adsorption of proteins due to a hydration layer formation, increasing the colloidal stability of SiO_2_NPs in a biological medium and exposing the groups for cellular recognition. Before assessing targeting ability, the colloidal stability of functionalized SiO_2_NPs was evaluated both in a cell culture medium supplemented with FBS and in human plasma. According to the size distribution curves (Figure 2b,c,d), both non-functionalized and functionalized SiO_2_NPs (0.5 mg·mL^–1^) remained stable in DMEM supplemented with 10% FBS after a 24 h incubation period, showing only a subtle aggregation. Therefore, it is impossible to conclude that ZW promoted the colloidal stability of NPs since the non-functionalized SiO_2_NPs were also colloidally stable under these conditions. Although aggregation was not verified, proteins can be adsorbed on SiO_2_NPs, which was determined by BCA assay and SDS-PAGE (Figure 2e and Supporting Information File 1, Figure S6). According to the BCA and SDS-PAGE results, more proteins are adsorbed on non-functionalized NPs than on functionalized ones.

The results in human plasma showed that non-functionalized SiO_2_NPs (Figure 2f) and SiO_2_NPs-ZW-FO (Figure 2h) were relatively stable for at least 24 h of incubation, as seen for the NPs incubated with DMEM. However, results for SiO_2_NPs-ZW (Figure 2g) showed an increase in the degree of aggregation, which was not verified in the incubation in DMEM. The BCA assay confirmed that more proteins are adsorbed on non-functionalized NPs than on functionalized ones, similar to the results in DMEM (10% FBS) (Figure 2i).

The difference between the NP stability in DMEM (10% FBS) and in plasma may be associated with the composition of the media. Then, LC-MS/MS technique was used to identify the protein corona composition. A list of the 20 most abundant proteins was obtained for DMEM (10% FBS) and functionalized and non-functionalized SiO_2_NPs (Supporting Information File 1, Table S1 and Supporting Information File 2). Albumin was the major protein identified in pure DMEM (10% FBS); however, there was a significant reduction in the amount of this protein in the hard corona of SiO_2_NPs, especially non-functionalized ones (Figure 2j). Although albumin is present in large quantities in FBS, proteins with different molecular weights can be adsorbed depending on their affinity to the NP surface, allowing less abundant proteins to be identified [42]. According to the results, while non-functionalized SiO_2_NPs presented the lowest albumin concentration in the corona, the addition of ZW promoted an increase in the amount of this protein in the hard corona. It is noteworthy that albumin is a dysopsonin, which means that the circulation time of NP is extended in case the protein corona is enriched with this protein [43–44]. Furthermore, variations were observed in the identification of the complement system and acute phase proteins when comparing non-functionalized SiO_2_NPs with functionalized ones. In particular, adding ZW promoted decreased absorption of such proteins (Figure 2j). Complement factors and acute phase proteins (e.g., C3 and alpha-1-antiproteinase) are called opsonins, so when present in high amounts in the protein corona they will induce macrophage recognition and engulfment of NPs, leading to the fast removal of NPs out of the body [43,45]. Finally, it should be noted that alpha and beta hemoglobins, classified in the group of other proteins, were also found in great intensity in the corona of SiO_2_NPs, especially in SiO_2_NPs-ZW. Such proteins are responsible for increasing the circulation time of NPs in the blood. Thus, it is concluded that adding the stabilizing group promotes an increase in dysopsonins and a decrease in opsonins.

The LC-MS/MS technique was also used for the identification of proteins after incubation of SiO_2_NPs in human plasma (Figure 2k). A list of the 20 most abundant proteins was obtained for human plasma and functionalized and non-functionalized SiO_2_NPs (Supporting Information File 1, Table S2 and Supporting Information File 3). Contrary to DMEM incubation, human plasma albumin and acute phase proteins were barely identified in the hard corona of functionalized and non-functionalized SiO_2_NPs. However, a large amount of apolipoproteins was identified in all cases, especially in the functionalized ones. Apolipoproteins are also dysopsonins, functioning to increase the circulation time of NPs in the body [43]. Coagulation factors (e.g., fibrinogen) constitute a significant portion of the corona on SiO_2_NPs, particularly in non-functionalized SiO_2_NPs. The addition of ZW resulted in a reduction of these proteins, which is crucial for applying these NPs in nanomedicine, as such proteins contribute to NPs clearance from the body, as mentioned earlier. While ZW had a favorable effect by increasing the amount of dysopsonins and decreasing opsonins, it also contributed to the aggregation of SiO_2_NPs-ZW in plasma. One hypothesis for this decreased stability is the greater adsorption of proteins from the complement system compared to non-functionalized NPs. However, it is worth noting that after adding the folate group, a significant reduction in this protein adsorption was observed, which could explain the stability of the dual-functionalized particles in human plasma. Statistical analysis of protein enrichment and depletion on SiO_2_NPs, SiO_2_NPs-ZW, and SiO_2_NPs-ZW-FO in relation to DMEM and human plasma (Figure 2l) clearly demonstrated these trends. For example, a decrease in the amount of serum albumin in the protein corona was observed compared to isolated media. Furthermore, there was a decrease in the identification of C3 and alpha-1-antiproteinase protein in the corona of functionalized SiO_2_NPs concerning non-functionalized SiO_2_NPs when incubated in DMEM (10% FBS). Additionally, there was a decrease in the amount of fibrinogen for the functionalized SiO_2_NPs when incubated in human plasma.

The results mentioned above reveal that ZW enabled the maintenance of colloidal stability of NPs in DMEM. Regarding human plasma, despite the formation of aggregates, the subsequent addition of folate ensured the stability of NPs in this medium. Moreover, in both evaluated media, there was a decrease in protein adsorption after the addition of ZW, primarily in the adsorption of opsonin.

### Hemolysis and cell viability assays

**Figure 3:**
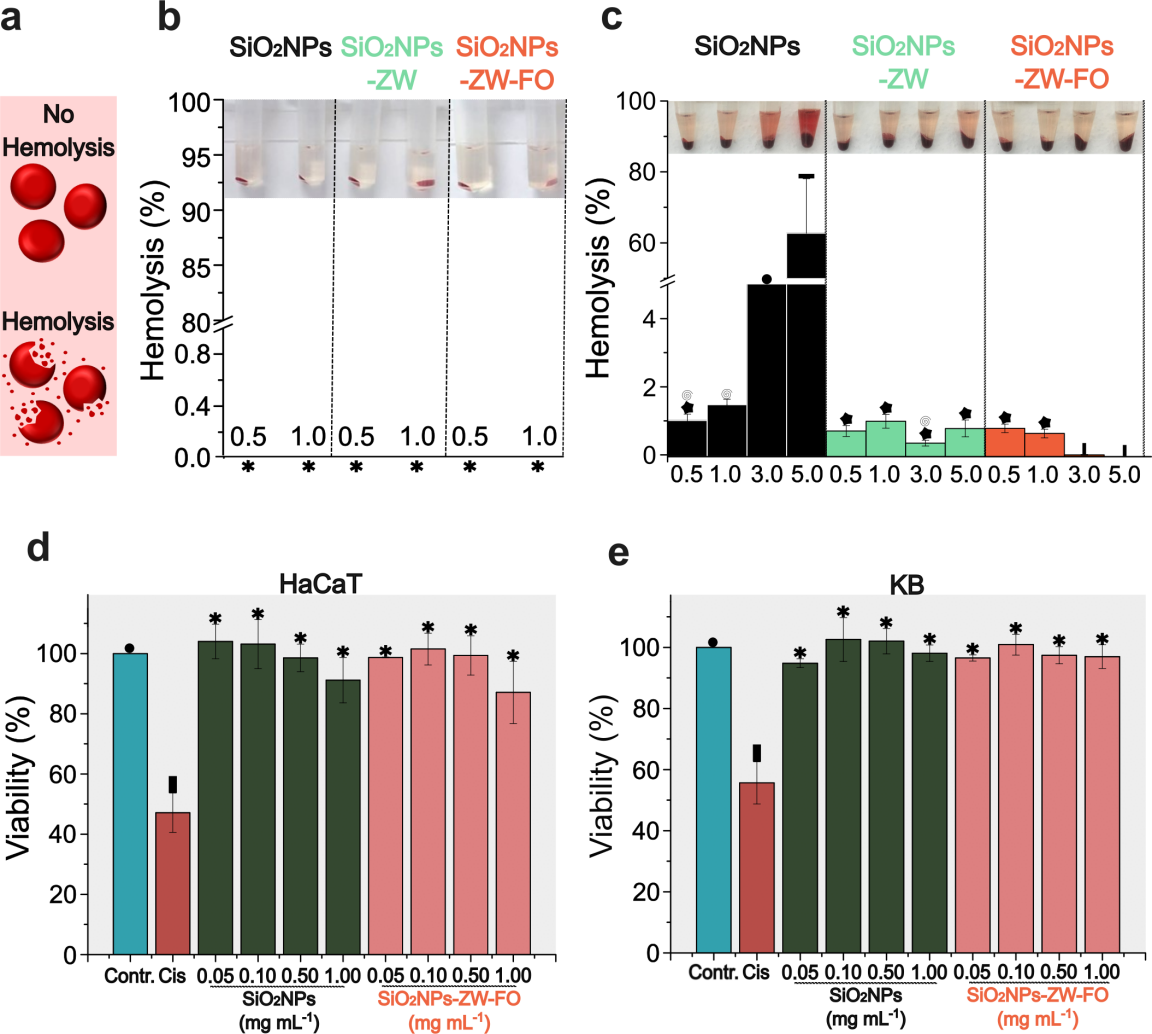
a) Illustration of intact red blood cells after the hemolysis process. Hemolytic activity of SiO_2_NPs, SiO_2_NPs-ZW, and SiO_2_NPs-ZW-FO diluted in b) DMEM (10% FBS) and c) murine blood. The concentrations of NPs used were 0.5 and 1.0 mg·mL^–1^ (b) and 0.5, 1.0, 3.0, and 5.0 mg·mL^–1^ (c). Results are presented as mean ± standard deviation (*n* = 3 for b and *n* = 10 for c). The common symbols on the top of the bars indicate that there is no statistical difference between them. For samples marked with an asterisk, no significant hemolysis was observed. Cell viability assay (Alamar Blue) of d) HaCat and e) KB cells after incubation for 24 h with non-functionalized SiO_2_NPs and with SiO_2_NPs-ZW-FO at concentrations of 0.05, 0.10, 0.50 and 1.00 mg·mL^–1^. Cisplatin (Cis) was added as a cell death control. Results are presented as mean ± standard deviation (*n* = 3). The common symbols on the top of the bars indicate that there is no statistical difference between them.

Considering the reduction in proteins adsorbed on SiO_2_NPs containing ZW, a longer blood circulation time for these particles is expected, favoring hemolytic activity (Figure 3a) [46]. Therefore, hemolysis assay is essential to determine the safe use of SiO_2_NPs in vivo. Experiments were conducted using SiO_2_NPs diluted in PBS and incubated with packed red blood cells and murine blood. According to the results, while the non-functionalized SiO_2_NPs exhibited a high hemolysis rate, the functionalized SiO_2_NPs showed no significant hemolytic activity when incubated in PBS (Supporting Information File 1, Figure S7). The hypothesis regarding the hemolytic activity of non-functionalized SiO_2_NPs involves their capability to generate reactive species of oxygen, along with electrostatic interactions of deprotonated silanol groups with membrane proteins and tetra alkyl ammonium groups, which are also present in red blood cell membranes [47–50]. The addition of the ZW compound possibly prevents these interactions, ensuring the colloidal stability of NPs without increasing hemolysis. It is noteworthy that for a material to be considered hemolytic, it must cause a percentage of hemolysis greater than 5% [36]. When the incubation was performed in DMEM (10% FBS), no hemolytic activity was observed, even for the non-functionalized NPs (Figure 3b). According to the literature [51–52], the protein corona suppresses hemolytic activity, as proteins induce modifications on the surfaces of NPs, altering the interactions between NPs and blood cells.

When the functionalized SiO_2_NPs were incubated in murine blood, the percentage of hemolysis was less than 5%, regardless of the concentration (Figure 3c). This result was similar to those obtained with SiO_2_NPs incubated in DMEM (10% FBS) and exposed to red blood cells. Conversely, non-functionalized SiO_2_NPs were hemolytic at concentrations of 3.0 and 5.0 mg·mL^–1^. Theoretically, since these SiO_2_NPs do not have surface functionality, it is expected to have a low hemolytic capacity due to the protein corona formation, as observed for concentrations of 0.5 and 1.0 mg·mL^–1^. One hypothesis for the increased hemolytic capacity of non-functionalized SiO_2_NPs at concentrations of 3.0 and 5.0 mg·mL^–1^ is the high particle concentration. The increase in NP concentration can reduce the density of proteins on the surface, exposing negative charges on the particles, which then interact with the red blood cells, leading to their rupture.

Regarding cell viability assays, both non-functionalized and functionalized SiO_2_NPs did not affect the viability, regardless of the evaluated concentration (Figure 3d,e). Thus, the results support the use of SiO_2_NPs for tests that gradually mimic the real conditions of the human body.

### Folate receptor expression and internalization of SiO_2_NPs in healthy and tumor cells

**Figure 4:**
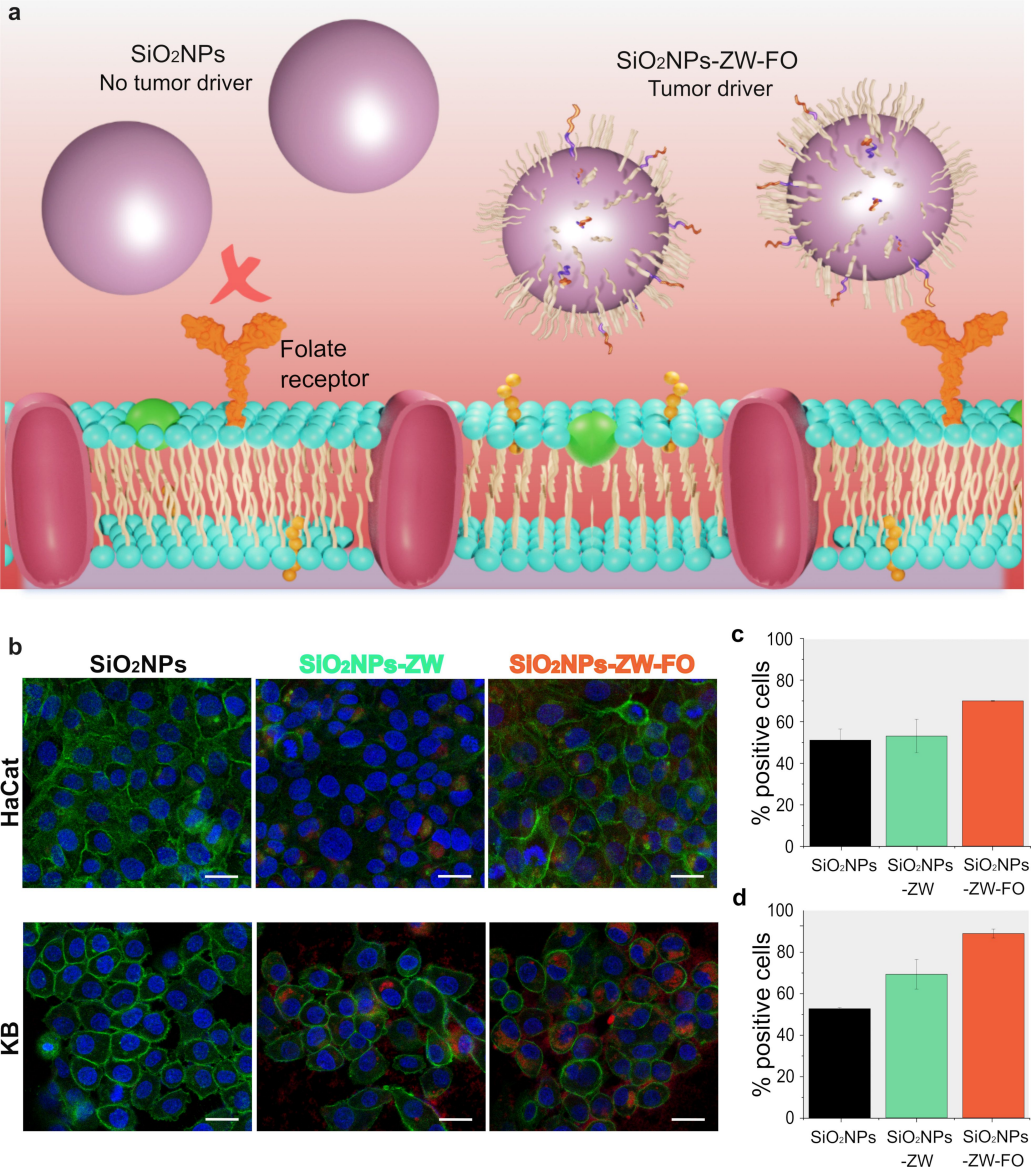
a) Representation of cellular recognition of SiO_2_NPs-ZW-FO through the folate receptor. b) Cell targeting assay using non-functionalized SiO_2_NPs and SiO_2_NPs-ZW-FO, after 24 h of incubation, obtained by an Operetta microscope. Cells were stained with DAPI (cell nucleus, blue) and phalloidin 488 (actin filaments, green) and SiO_2_NPs can be visualized in red. Scale bar: 25 μm. Percentage of positive cells for SiO_2_NPs, SiO_2_NPs-ZW, and SiO_2_NPs-ZW-FO, where c) HaCat and d) KB cells.

To confirm that SiO_2_NPs-ZW-FO are more recognized by cells with high folate receptor expression than non-functionalized SiO_2_NPs, both were added to 2D cell cultures to evaluate their targeting ability (Figure 4a). The cell lines HaCat (healthy cell, low folate receptor expression) and KB (tumor cell, high folate receptor expression) were chosen because they are a healthy and a tumor cell line, respectively, and have differences in folate receptor expression (Supporting Information File 1, Figure S8).

According to the images (Figure 4b), SiO_2_NPs-ZW-FO were considerably more recognized by tumor cells, KB, than SiO_2_NPs-ZW and non-functionalized SiO_2_NPs after 24 h of incubation. Furthermore, the recognition of SiO_2_NPs-ZW-FO in healthy cells, HaCat, was lower than that observed in tumor cells. This result is justified by the high expression of the folate receptor on the surface of KB cells, which increases the probability of receptor binding to the tumor driver on the surface of SiO_2_NPs-ZW-FO. Importantly, similar results were obtained for incubation times of 3, 6, and 12 h (Supporting Information File 1, Figure S9).

The flow cytometry technique was further used to quantitatively assess the internalization of NPs in both healthy and tumor cells. For this technique, SiO_2_NPs, SiO_2_NPs-ZW, and SiO_2_NPs-ZW-FO were utilized. Initially, it was observed that functionalized and non-functionalized NPs were more internalized in tumor cells than in healthy cells after 24 h of incubation (Figure 4c,d). Moreover, SiO_2_NPs-ZW-FO were more internalized by tumor cells compared to non-functionalized NPs. A similar trend was observed for the experiments conducted within 3 h of incubation (Supporting Information File 1, Figure S10). It is important to highlight that the stabilizer group (ZW) contributed to the cell internalization process even without the addition of the folate group. This result aligns with existing literature, which suggests that the density and type of zwitterionic groups, as well as the charge of the NP, can influence the protein corona composition and potentially enhance cellular internalization [53–54].

## Conclusion

Herein, we successfully developed dually functionalized SiO_2_NPs, which exhibit stability in complex media and possess an efficient tumor-targeting group. We performed experiments on colloidal stability, protein corona formation, toxicity, and tumor targeting in biologically relevant media, including supplemented cell culture media, human plasma, and murine blood. Our results indicated that (i) low amounts of human plasma proteins adsorb on doubly functionalized SiO_2_NPs, (ii) the addition of ZW leads to a decrease in the adsorption of opsonins, which are recognized by the immune system, (iii) these functionalized particles remain colloidally stable in complex media, (iv) they exhibit no hemolytic behavior, and (v) they are more readily internalized by tumor cells than by healthy ones. Such experiments are of pivotal relevance for obtaining more reliable results and open possibilities for developing NPs that are increasingly efficient and suitable for medical applications. Ensuring that a platform efficiently functions for in vitro assays is a crucial step in predicting in vivo effects. Our system proves to be a successful model that can be extended to other types of NPs, such as degradable mesoporous silica nanoparticles, which are highly porous and can degrade under specific biological conditions, thus preventing inconvenient accumulation in the body.

## Supporting Information

^1^H NMR spectrum for ZW compound; a scheme for functionalization of fluorescent SiO_2_NPs; a schematic representation of immunoprecipitation assay; high-resolution XPS spectrum for functionalized and non-functionalized SiO_2_NPs; analytical curve of fluorescence responses as a function of SiO_2_NPs concentration; analysis of protein corona formation after incubation of NPs in DMEM and human plasma (SDS-PAGE and LC-MS assays); hemolysis assay for functionalized and non-functionalized SiO_2_NPs; identification of the folate receptor in HaCat and KB cells; cell targeting assay using non-functionalized SiO_2_NPs and SiO_2_NPs-ZW-FO.

File 1 Additional figures and tables.

File 2 Mass spectrometry proteomic data (raw and processed data, DMEM).

File 3 Mass spectrometry proteomic data (raw and processed data, plasma human).

## Data Availability

All data that supports the findings of this study is available in the published article and/or the supporting information to this article.
