# Supplementary material for: Dual-functionalized architecture enables stable and tumor cell-specific SiO2NPs in complex biological fluids
Source: Beilstein J Nanotechnol. 2024 Oct 7;15:1238–52. doi: 10.3762/bjnano.15.100 (PMC11472657; doi:10.3762/bjnano.15.100)
Supplement: File 1 — Additional figures and tables. [file Beilstein_J_Nanotechnol-15-1238-s001.pdf]

BEILSTEIN JOURNAL OF NANOTECHNOLOGY

Supporting Information

for

Dual-functionalized architecture enables stable and tumor cell-specific SiO_2NPs in complex biological fluids

Iris Renata Sousa Ribeiro, Raquel Frenedoso da Silva, Romênia Ramos Domingues, Adriana Franco Paes Leme and Mateus Borba Cardoso

Beilstein J. Nanotechnol. **2024,** *15,* 1238–1252. doi:10.3762/bjnano.15.100

Additional figures and tables

License and Terms: This is a supporting information file under the terms of the Creative Commons Attribution License (https://creativecommons.org/licenses/by/4.0). Please note that the reuse, redistribution and reproduction in particular requires that the author(s) and source are credited and that individual graphics may be subject to special legal provisions.
The license is subject to the *Beilstein Journal of Nanotechnology* terms and conditions: (https://www.beilstein-journals.org/bjnano/terms)

1. Characterization of ZW

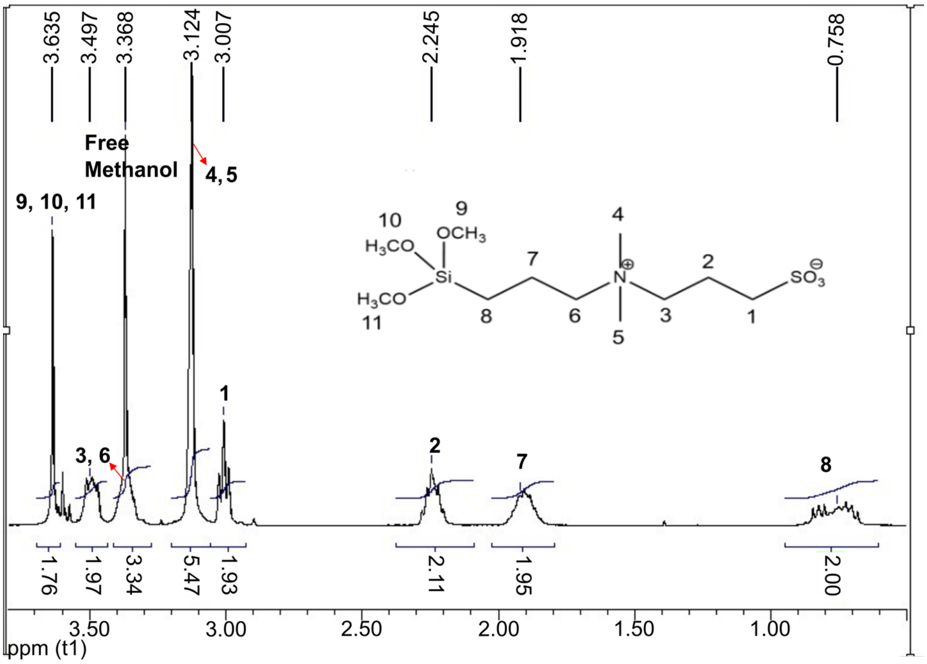

Figure S1: ^{1}H NMR spectrum for ZW compound. Band assignments are included in the spectrum.

2. Chemical Structures

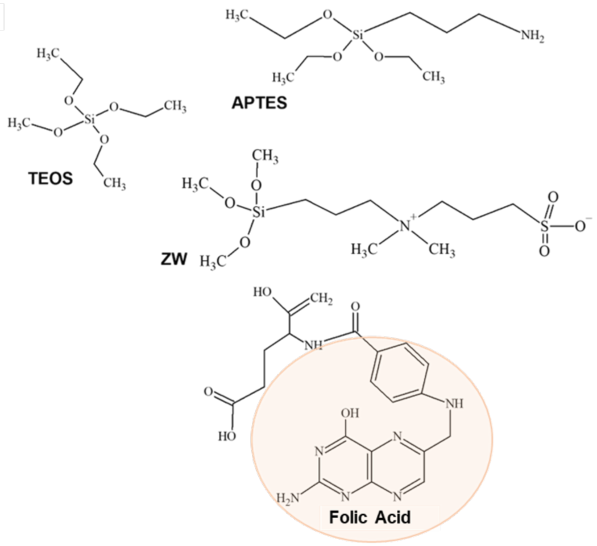

Figure S2: Chemical structures of the main compounds used for the synthesis and functionalization of SiO_2NPs. In the structure of folic acid, the region that will be recognized by the folate receptor is highlighted.

3. Immunoprecipitation assay

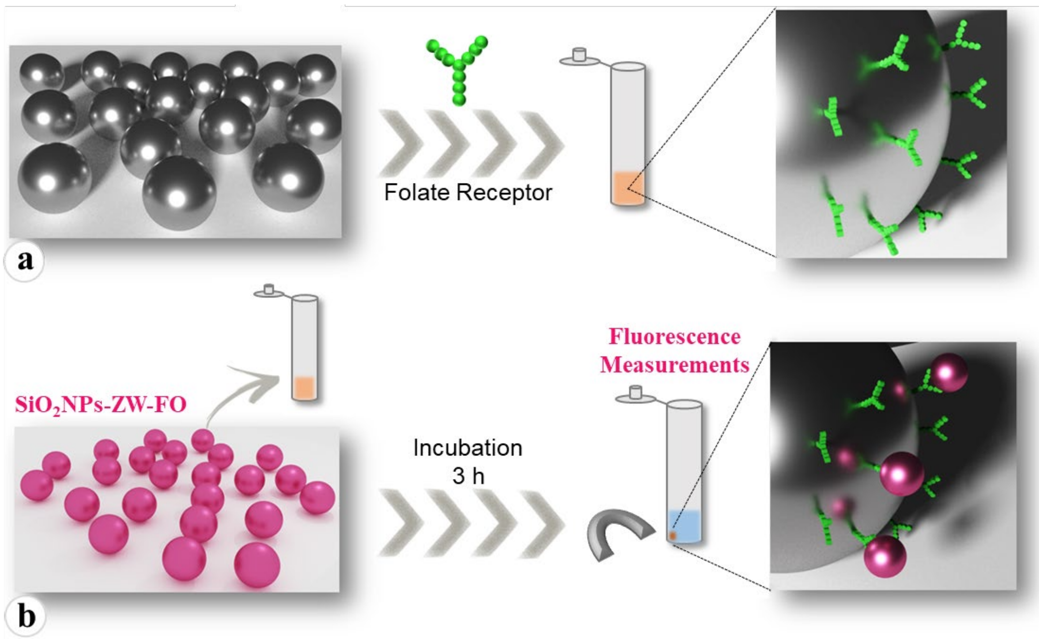

Figure S3: Schematic representation of the magnetic beads preparation for immunoprecipitation assay. Briefly, the beads are modified with a) the folate receptor and subsequently exposed to b) SiO_2NPs and SiO_2NPs-ZW-FO. After incubation, measurements are performed using the fluorescence technique.

4. Nanoparticle characterization

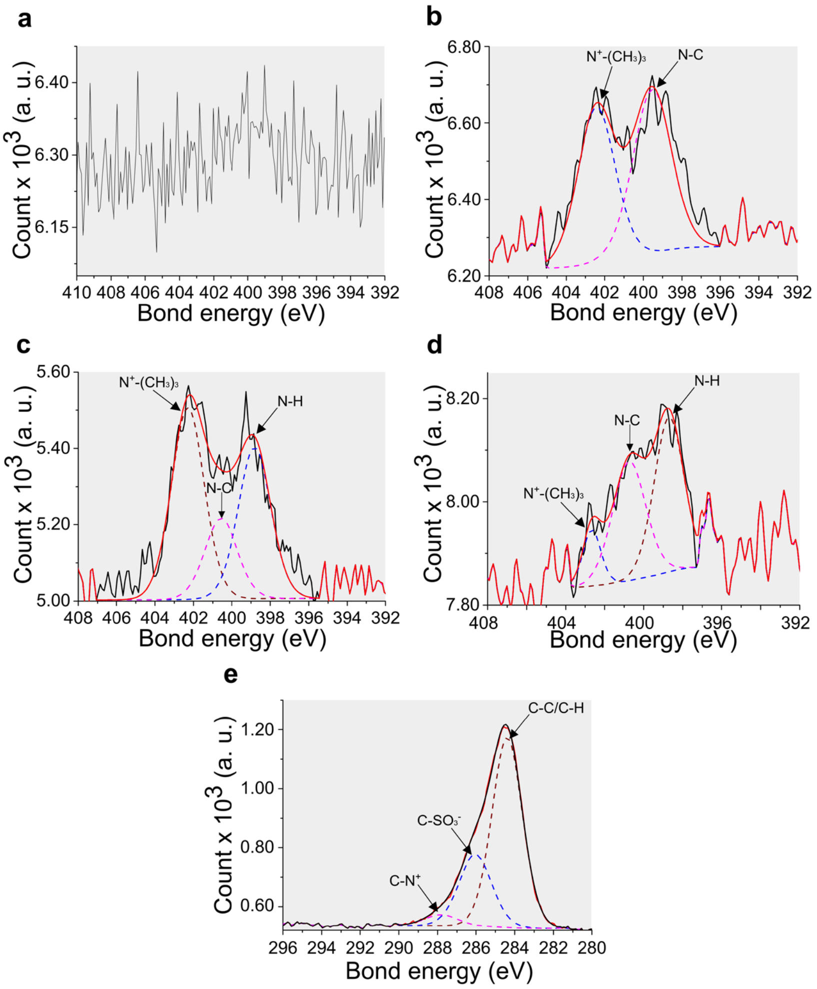

Figure S4: High resolution XPS spectrum (N 1s) for a) SiO_2NPs, b) SiO_2NPs-ZW, c) SiO_2NPs-ZW-NH_2, d) SiO_2NPs-ZW-FO. High resolution XPS spectrum (C 1s) for e) SiO_2NPs-ZW-NH_2. The main peaks for each sample are marked on the spectrum itself.

5. Nanoparticle characterization

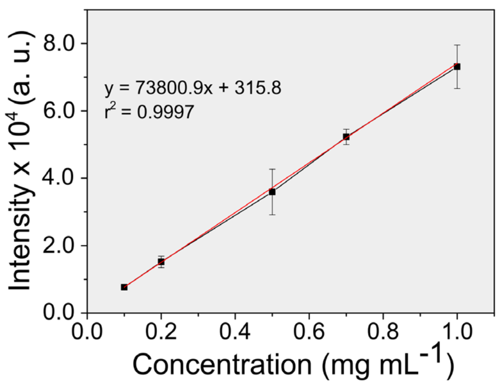

Figure S5: Analytical curve showing fluorescence responses as a function of SiO_2NPs concentration (0.1, 0.2, 0.5, 0.7 and 1.0 mg·mL^{-1}).

6. Protein corona formation

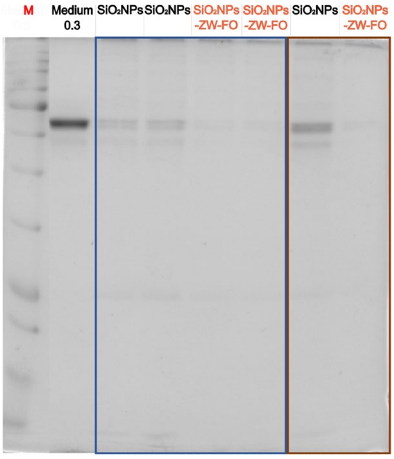

Figure S6: Polyacrylamide gel electrophoresis of non-functionalized and functionalized SiO_2NPs samples after incubation in DMEM medium (10% FBS). The concentrations of SiO_2NPs (functionalized and non-functionalized) used were 2 $mg \cdot mL^{-1}$ (samples delimited by the blue square, duplicates for each sample) and 5 $mg \cdot mL^{-1}$ (samples delimited by the orange square). DMEM medium (10% FBS) was also used for control without NPs at 0.3 dilution. Lane M is a protein marker.

7. Protein corona composition

Table S1: 20 Most abundant proteins identified in DMEM and hard corona of SiO_2NPs, SiO_2NPs-ZW, and SiO_2NPs-ZW-FO in descending order.

DMEM	SiO_2NPs	SiO_2NPs-ZW	SiO_2NPs-ZW-FO
Albumin	Complement C3	Albumin	Albumin
Alpha-1-antiproteinase	Hemoglobin fetal subunit beta	Trypsin	Similar to complement C4-A precursor
Alpha-2-HS-glycoprotein	Apolipoprotein A-I	Gelsolin	Apolipoprotein A-I
Serotransferrin	Albumin	Similar to complement C4-A precursor	Trypsin
Alpha-2-macroglobulin	Hemoglobin subunit alpha	Hemoglobin fetal subunit beta	Alpha-2-HS-glycoprotein
Hemoglobin fetal subunit beta	Alpha-1-antiproteinase	Alpha-2-HS-glycoprotein	Gelsolin
Fetuin-B	Gelsolin	Apolipoprotein A-I	Hemoglobin fetal subunit beta
Alpha-fetoprotein	Alpha-2-HS-glycoprotein	Complement C3	Complement C3
Vitamin D-binding protein	Similar to complement C4-A precursor	Methanethiol oxidase	Hemoglobin subunit alpha
Complement C3	Trypsin	Alpha-1-antiproteinase	Alpha-1-antiproteinase
Hemoglobin subunit alpha	Complement factor B	Hemoglobin subunit alpha	Tetranectin
Apolipoprotein A-I	Alpha-2-macroglobulin	Complement factor H	Complement factor H
Trypsin	Inter-alpha-trypsin inhibitor heavy chain H2	Tetranectin	Kininogen-1
Inter-alpha-trypsin inhibitor heavy chain H2	Alpha-fetoprotein	Complement factor B	Alpha-2-macroglobulin
Hemopexin	Plasminogen	Alpha-2-macroglobulin	Complement factor B
Methanethiol oxidase	Inter-alpha-trypsin inhibitor heavy chain H4	Thrombospondin-1	Inter-alpha-trypsin inhibitor heavy chain H2
Serpin A3-1	Fetuin-B	Plasminogen	Inter-alpha-trypsin inhibitor heavy chain H4
Antithrombin-III	Actin	Pigment epithelium-derived factor	Thrombospondin-1
Similar to complement C4-A precursor	Complement factor H	Plasminogen	Apolipoprotein E
Elongation factor 1-alpha	Apolipoprotein B-48	Apolipoprotein E	Pigment epithelium-derived factor

Table S2: 20 Most abundant proteins identified in human plasma and hard corona of SiO_2NPs, SiO_2NPs-ZW, and SiO_2NPs-ZW-FO in descending order.

PLASMA	SiO_2NPs	SiO_2NPs-ZW	SiO_2NPs-ZW-FO
Albumin	Apolipoprotein A-I	Apolipoprotein B-100	Apolipoprotein B-100
Apolipoprotein A-I	Fibrinogen alpha chain	Apolipoprotein A-I	Apolipoprotein A-I
Serotransferrin	Fibrinogen gamma chain	Histidine-rich glycoprotein	Fibrinogen alpha chain
Alpha-1-antitrypsin	Fibrinogen beta chain	Fibrinogen alpha chain	Fibrinogen gamma chain
Immunoglobulin kappa	Apolipoprotein B-100	Complement C1q subcomponent subunit C	Fibrinogen beta chain
Fibrinogen alpha chain	Albumin	Complement C1q subcomponent subunit B	Apolipoprotein E
Haptoglobin	Immunoglobulin heavy constant gamma 1	Apolipoprotein E	Immunoglobulin heavy constant gamma 1
Apolipoprotein B-100	Histidine-rich glycoprotein	Trypsin	Histidine-rich glycoprotein
Fibrinogen gamma chain	Apolipoprotein E	Immunoglobulin heavy constant gamma 1	Immunoglobulin kappa
Immunoglobulin lambda constant 3	Apolipoprotein A-II	Immunoglobulin kappa	Trypsin
Immunoglobulin heavy constant alpha 1	Immunoglobulin kappa	Fibrinogen gamma chain	Albumin
Fibrinogen beta chain	Sialic acid-binding Ig-like lectin	Complement C1q subcomponent subunit A	Complement C1q subcomponent subunit C
Immunoglobulin heavy constant gamma 1	Serum amyloid A-4	Albumin	Immunoglobulin lambda constant 3
Apolipoprotein A-IV	Trypsin	Fibrinogen beta chain	Complement C1q subcomponent subunit B
Vitamin D-binding protein	Gelsolin	Immunoglobulin lambda constant 3	Serum amyloid A-4
Transthyretin	Immunoglobulin lambda constant 3	Plasma kallikrein	Complement C1q subcomponent subunit A
Alpha-1-antichymotrypsin	Complement C1q subcomponent subunit C	Immunoglobulin heavy constant mu	Complement C3
Trypsin	Complement C1q subcomponent subunit B	Complement C3	Apolipoprotein A-II
Alpha-2-macroglobulin	Complement C3	Kininogen-1	Sialic acid-binding Ig-like lectin
Plasma protease C1 inhibitor	Apolipoprotein A-IV	Immunoglobulin heavy constant gamma 3	Immunoglobulin heavy constant mu

8. Hemolysis assay

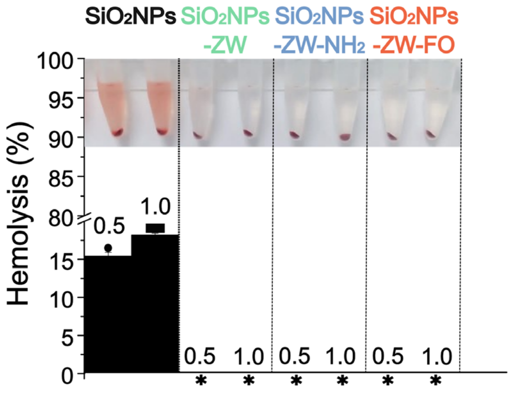

Figure S7: Hemolytic activity of SiO_2NPs, SiO_2NPs-ZW, SiO_2NPs-ZW-NH_2, and SiO_2NPs-ZW-FO diluted in PBS 1×. The concentrations of NPs used were 0.5 and 1.0 mg·mL^{-1}. Results are presented as mean ± standard deviation (n = 3). The common symbols on the top of the bars indicate that there is no statistical difference between them. For samples marked with an asterisk, no significant hemolysis was observed.

9. Folate receptor expression

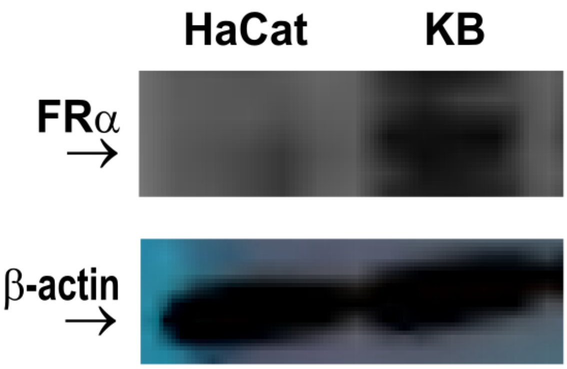

Figure S8: Western blot of HaCat and KB cell lysates (20 µg of protein), showing identification of the folate receptor (37 kDa) by the anti-folate receptor alpha antibody. FRα: Folate alpha receptor. As expected, the band referring to FRα in the KB cell was more intense than the one in the HaCat cell, indicating a higher expression of the receptor. β-actin protein (42 kDa) was used as a loading control.

10. Nanoparticle internalization

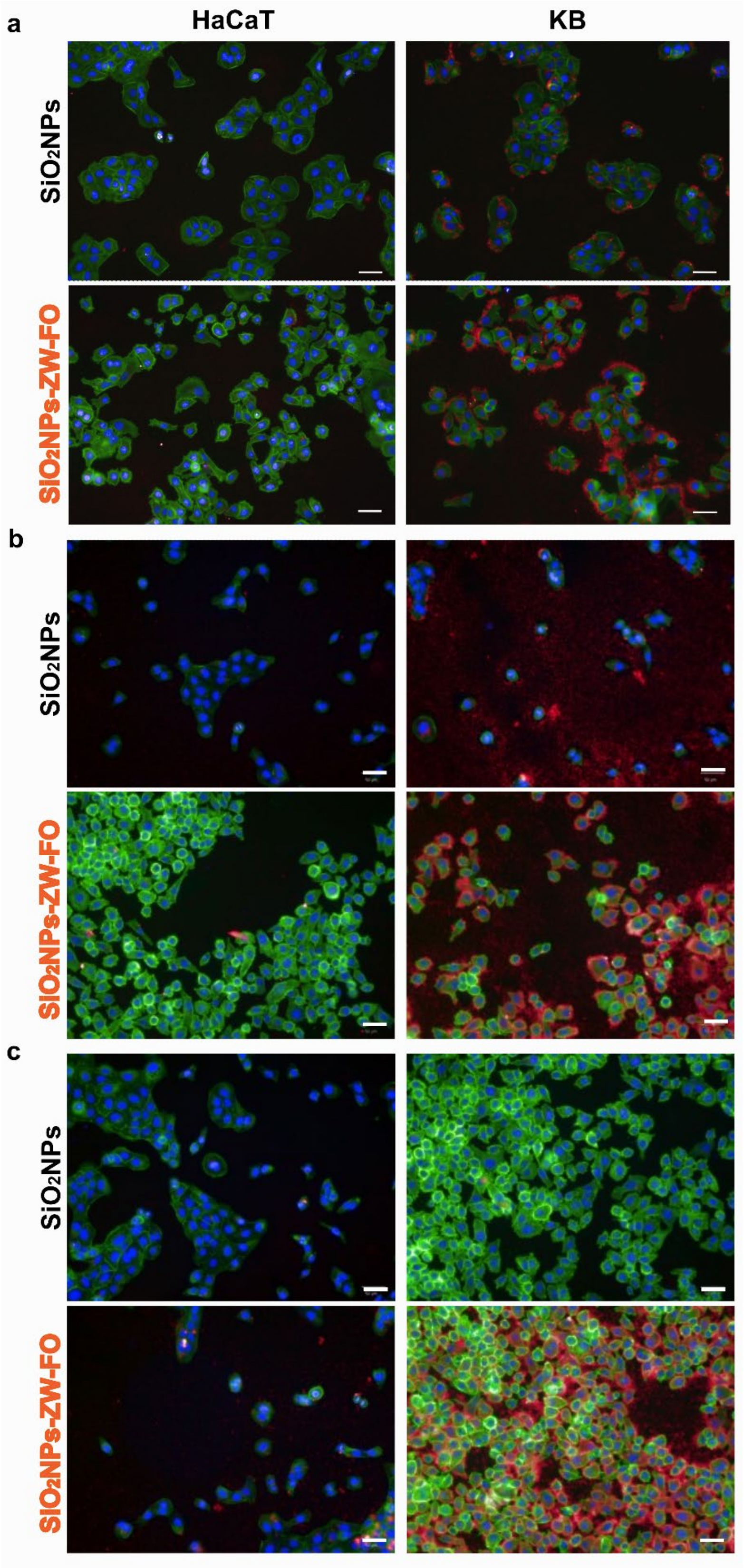

Figure S9: Cell targeting assay using non-functionalized SiO_2NPs and SiO_2NPs-ZW-FO, after a) 3 h, b) 6 h, and c) 12 h of incubation, obtained by an Operetta microscope. Cells were stained with DAPI (cell nuclei, blue) and phalloidin 488 (actin filaments, green), and SiO_2NPs can be visualized in red. Scale bar: 50 μm.

11. Nanoparticle internalization

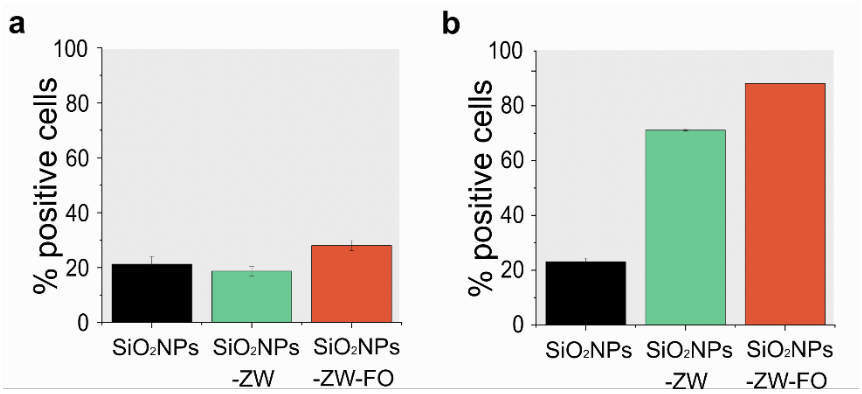

Figure S10: Percentage of positive cells for SiO_2NPs, SiO_2NPs-ZW, and SiO_2NPs-ZW-FO, where a) HaCat and b) KB cells.
